# Associations between Gastrointestinal Nematode Infection Burden and Lying Behaviour as Measured by Accelerometers in Periparturient Ewes

**DOI:** 10.3390/ani12182393

**Published:** 2022-09-13

**Authors:** Eiry Gwenllian Williams, Chelsea N. Davis, Manod Williams, Dewi Llyr Jones, David Cutress, Hefin Wyn Williams, Peter M. Brophy, Michael T. Rose, Rebekah B. Stuart, Rhys Aled Jones

**Affiliations:** 1 Institute of Biological, Environmental and Rural Sciences (IBERS), Aberystwyth University, Aberystwyth SY23 3DA, UK; 2 Tasmanian Institute of Agriculture, University of Tasmania, Sandy Bay, TAS 7005, Australia; 3 Hybu Cig Cymru, Aberystwyth SY23 3FF, UK

**Keywords:** precision livestock farming, sheep, periparturient ewes, gastrointestinal nematodes, accelerometers, lying behaviour

## Abstract

**Simple Summary:**

Novel sensor technologies have great potential to improve animal health and welfare on farms by identifying disease early in livestock. These technologies are yet to be widely applied in sheep flocks despite their great potential to aid control of costly disease such as those caused by parasitic infection. In this study, leg-attached accelerometer sensors recorded the behaviour of 54 ewes in late pregnancy, with the aim of discovering if gastrointestinal nematode (GIN) infection levels were associated with behavioural variation. It was found that ewes laid down more often on average when infected with increasing numbers of GIN. Each lying bout was also shorter in length on average in ewes infected with higher levels of GIN. The results demonstrate that ewe behaviour can be an indication of parasite infection levels, and thus automated monitoring of sheep behaviour could allow animals to be treated efficiently against GIN in the future, maximising animal health and minimising production losses.

**Abstract:**

The application of precision livestock farming (PLF) technologies will underpin new strategies to support the control of livestock disease. However, PLF technology is underexploited within the sheep industry compared to other livestock sectors, and research is essential to identify opportunities for PLF applications. These opportunities include the control of endemic sheep disease such as parasitic gastroenteritis, caused by gastrointestinal nematode infections, which is estimated to cost the European sheep industry EUR 120 million annually. In this study, tri-axial accelerometers recorded the behaviour of 54 periparturient Welsh Mule ewes to discover if gastrointestinal nematode (GIN) infection burden, as measured by faecal egg count (FEC), was associated with behavioural variation. Linear mixed models identified that increasing FECs in periparturient ewes were significantly associated with a greater number of lying bouts per day and lower bout durations (*p* = 0.013 and *p* = 0.010, respectively). The results demonstrate that FECs of housed periparturient ewes are associated with detectable variations in ewe behaviour, and as such, with further investigation there is potential to develop future targeted selective treatment protocols against GIN in sheep based on behaviour as measured by PLF technologies.

## 1. Introduction

Precision livestock farming (PLF) technologies are increasingly being developed and applied to monitor animal behaviour, with the aims of optimising animal health management and diagnosing disease earlier in order to implement informed treatment strategies [1]. PLF technologies have been applied to the dairy, pig and poultry sectors, where their use has led to increased production and welfare [2]. However, PLF in the sheep sector has not been as widely investigated or applied, and further research is urgently needed to explore the viability of PLF in sheep flocks, with the aim being to enhance sheep health and welfare and the economic viability of the industry [3]. 

Gastrointestinal nematodes (GIN) are estimated to cost the European sheep industry EUR 120 million annually, impacting sheep health and productivity [4]. GINs are controlled by anthelmintics; however, due to GIN anthelmintic resistance (AR), controlling GIN in flocks has become challenging [5]. One of the key strategies to mitigate AR and to further reduce AR development is to decrease reliance on anthelmintics by ensuring a correct diagnosis is made prior to treatment, a strategy that must be applied rapidly due to continued excessive anthelmintic use [6,7]. One method of decreasing anthelmintic use whilst continuing to control GIN effectively is through targeted selective treatment (TST), a strategy where only animals in need of treatment are medicated [8,9]. TST has been shown to reduce anthelmintic use in lambs, ewes, and cattle, leading to reduced AR development without detrimental effects on productivity [7,10,11,12].

Recent research has identified various physical measures, such as weight, daily live weight gain (DLWG), and body condition score (BCS) as suitable proxies to determine if treatment against GIN infections is required in sheep [7,9,11]. However, the use of physical examination as a treatment proxy is not optimal, as these physical signs are only visible after sheep exhibit decreased performance [13,14,15]. Changes in animal behaviour may be the first indicator of disease [16]. Considering that associations between parasitic infections and animal behaviour have previously been observed [17], there may be potential to use PLF technologies such as accelerometers, which can classify animal behaviour, to record and evaluate an individual animal’s behaviour in relation to GIN infection status [18,19]. 

Behaviour monitoring of lambs [18,20] and cattle [21,22] using accelerometers and GPS trackers has demonstrated relationships between behaviour and their respective GIN burdens. However, studies using PLF technologies to monitor the behaviour of ewes in relation to GIN burden are limited, with there being no known studies focusing on periparturient ewes, which are extremely vulnerable to GIN infection. Therefore, the main aim of this study was to explore associations between periparturient ewe GIN burden, measured by faecal egg counts (FEC), and ewe lying behaviour during late pregnancy using tri-axial accelerometers. 

## 2. Materials and Methods

### 2.1. Data Collection

Ethical approval for this study was obtained from the Aberystwyth University Animal Welfare and Ethical Review Board on 18 December 2018. This study was conducted as part of a larger multi-flock study which focused on monitoring the behaviour of periparturient ewes in the final weeks of pregnancy [23]. Within this larger study, the behaviour of 98 housed Welsh Mule ewes was monitored at Aberystwyth University’s Gogerddan farm, Ceredigion, Wales. Full details regarding the larger multi-flock study have previously been reported by Williams et al. [23]. However, in brief, HOBO Pendant G accelerometers (Onset Computer Corporation, Bourne, MA, USA) were fitted to the right hind leg of each ewe to record ewe lying behaviour in late pregnancy. Accelerometers were fitted vertically (*X*-axis pointing upwards, *Z*-axis pointing to the left) and set to sample a position at 1-min intervals for a duration of 14 days. Ewes were housed with the wider flock and split across six straw-bedded pens measuring 8.8 m × 6.1 m. Before housing, ewes had been grazing permanent pastures where they had become naturally infected with GIN, whilst no anthelmintic treatment against GIN had been administered to these ewes for at least 6 months prior to the study. After lambing, ewes were moved into mothering pens, where they remained for 24 h before being turned out to pasture. 

In these mothering pens post-lambing, lambed ewes were visited each morning and evening throughout the study with the aim of collecting fresh faecal samples from the floor of individual pens. In total, fresh individual faecal samples from 54 ewes were successfully obtained. The collected faecal samples were immediately stored at 4 °C, and their FECs were measured using the Mini-FLOTAC method, which has a sensitivity of 5 EPG [24], within 48 h of collection. Here, 5 g of the faecal sample was mixed with 45 mL of a saturated salt solution in a Fill-FLOTAC. The slurry was then transferred from the Fill-FLOTAC into the two chambers of a Mini-FLOTAC disk and left to stand for 10 min. The Mini-FLOTAC reading disk was then rotated before eggs visible in both reading chambers were counted under ×100 magnification using a Leicia CME light microscope (Leicia Camera, Wetzlar, Germany). The total number of eggs in both chambers was multiplied by 5 to calculate an eggs per gram (EPG) value for each sample. Data regarding ewe age, BCS (scale = 1–5) [25] and litter size were recorded at the start of the study, whilst lamb birth weights were also noted.

### 2.2. Data Processing and Statistical Analysis

Data collected by the accelerometers (g-force values) for each ewe were processed using R [26] as described by Williams et al. [23]. In brief, raw accelerometer data were computed to determine ewe lying behaviour, which included mean number of daily lying bouts (n/d), mean daily lying bout duration (min/bout) and mean daily lying time (min/d) using the *X*-axis data only. The measures of behaviour were standardised by averaging 3 days of data from days -10, -9 and -8 pre-lambing for each ewe, as it was hypothesised that the behaviour of ewes would be later influenced by the onset of lambing [27,28], the fact that some ewes lambed -11 days pre-lambing, and as a minimum of 3 days of behaviour data has been found to be required for reliable estimates of cattle and goat behaviour [29,30]. 

All statistical analyses were undertaken in R [26]. Linear mixed models (LMM) were created using the glmmTMB function in R [31] to identify if ewe FEC post-lambing was associated with ewe behaviour during late pregnancy. In this analysis, a measure of behaviour, either mean number of bouts per day, mean daily lying time or mean bout duration, was the dependent variable, and ewe log_10_ transformed FEC was the independent variable. Other variables, including litter size, ewe BCS, ewe age and average lamb birth weight were also tested for their association with each ewe behavioural measure in multivariate models, which allowed their impact on ewe behaviour to be controlled for whilst assessing associations between log FEC and behaviour. Furthermore, pen was set as a random effect factor to account for possible variation between pens. 

Diagnostic tests were conducted to ensure that each LMM created in this study was appropriately fitted using the DHARMa package in R [32]. These diagnostic tests included Kolmogorov–Smirnov tests of standardised residual normality and tests of residual dispersion.

## 3. Results

The average behavioural measurements (number of daily lying bouts, lying bout duration and daily lying time), FEC counts, age and BCS of periparturient ewes, as well as their litter size and lamb birth weight are presented in Table 1. In the study, 59% of ewes carried twins and 41% carried singletons. All but one ewe in the study had a positive FEC, whereby at least 1 GIN egg was detected. Inclusion/exclusion of this negative ewe in the data analysed did not change overall findings.

**Figure 1:**
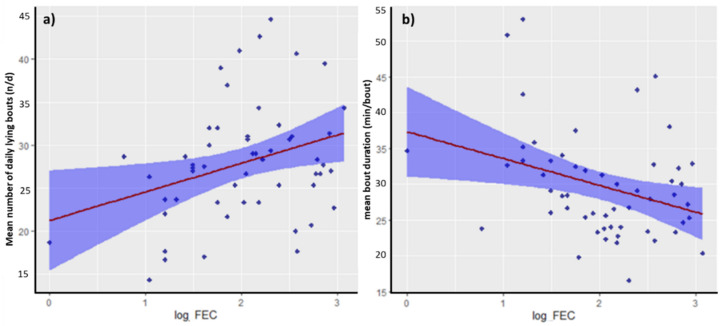
Visualisation of significant (*p* < 0.05) association detected by linear mixed models between log FEC and (**a**) mean number of daily lying bouts (n/d) and (**b**) mean bout duration (min/bout). Shaded areas signify 95% confidence intervals of the regression equation. Residual degrees of freedom = 50 for each model.

Log FEC was significantly associated with ewe mean number of daily lying bouts and mean lying bout duration (*p* = 0.013 and *p* = 0.010, respectively) (Table 2). Increasing FECs were associated with an increased number of lying bouts per day and a significantly shorter duration per bout, with regression coefficients demonstrating a trend where ewe mean number of lying bouts per day increased by 3.3 per unit increase in log FEC, with each lying bout 3.7 min shorter with each one unit increase in log FEC (Figure 1). Residual diagnostic tests revealed that these LMMs were appropriately fitted. There was no significant association between ewe log FEC and mean daily lying time (*p* > 0.05). None of the other recorded measures in this study (BCS, age, litter size, lamb birth weight) were significantly associated with any measure of ewe behaviour (*p* > 0.05), whilst both ewe mean number of daily lying bouts and mean lying bout duration remained significantly associated with log FEC when controlling for these other variables (*p* = 0.008 and *p* = 0.013, respectively) (Table 3). 

## 4. Discussion

In this study, FECs of periparturient ewes were shown to be significantly associated with ewe lying behaviour. Ewes that had higher FECs were observed to have an increased number of lying bouts per day on average, with each lying bout significantly shorter in length. For every unit increase in log FEC, ewe mean number of lying bouts per day increased by 3.3 on average, with each lying bout 3.7 min shorter on average. This indicates that ewes with higher FECs were increasingly restless. Similar findings were observed by Hogberg et al. [22] in first season grazing cattle, where steers exposed to a high level of GINs had a significantly higher number of lying bouts per day. Previous studies have also highlighted associations between GIN infection burden and sensor-measured behaviours in sheep; however, the directions of these associations were inconsistent. Hogberg et al. [33] recorded a significant relationship between GIN infection burden in lambs and their overall lying time per day, unlike our study, and did not find any significant relationship between GIN infection burden and measures of lying bouts. However, overall activity as measured by the motion index was significantly higher in lambs with higher GIN burdens, which may indicate that increasing GIN burdens are associated with lamb restlessness. In a study that tracked movements using GPS, Falzon et al. [18] found evidence of increased activity in heavily infected Merino sheep, which included an increase in their overall mean speed as their FEC increased. However, Grant et al. [34] found that sheep walked slower as FEC increased, whilst Ikurior et al. [20] recorded reduced activity in lambs that were untreated against GINs compared to those that were wormed. Burgunder et al. [35], meanwhile, found no significant association between standard measures of behaviour and GIN infection burden in ewes. 

Inconsistent associations between GIN infection burden and cattle behaviours have also been observed between studies and within studies [21,36]. For example, Hogberg et al. [36] investigated the effects of subclinical nematode parasitism on activity and rumination in first season grazing cattle, discovering that the mean daily steps and mean daily motion index were significantly higher in heavily infected cattle, but only during days 62–69 of the study. The study also found that cattle heavily infected with GIN had a higher mean daily lying time per day (+28.5 min) during the first 40 days on pasture [36]. The inconsistencies observed between these studies and our study are likely due to multiple factors, such as species (cattle vs. sheep), animal age and immunological status (lambs vs. ewes), physiology (pregnant vs. not pregnant), management (housed vs. grazing), GIN infection type (natural vs. experimental), infective GIN species composition and sensors used (accelerometer vs. GPS vs. qualitative behavioural assessment). Although PLF technologies have great potential as diagnostic tools for GIN infections in ruminants [33,36], the complexities of controlling multiple factors associated with animal behaviour may be a considerable challenge. To alleviate this issue, multivariate models could be utilised. Research using cattle has demonstrated that the inclusion of non-sensor data can significantly improve the performance of sensor-based mastitis detection systems [37]. 

Despite the inconsistencies observed, it is clear that ruminants heavily infected with GINs exhibit behavioural changes [20], although it is unclear why these changes occur. It has been suggested that the re-direction of resources within a parasitised animal towards producing an immune response can influence behaviour [34,35], as can general discomfort from pain and stress [22]. Animal behaviour in relation to general illness has been widely studied [38,39,40,41], although GIN-infection-influenced behavioural changes appear to vary significantly from the classical behavioural changes associated with other health issues, where a decline in activity is typically seen when an animal is ill [40]. This is likely due to lethargy associated with sickness behaviour, which is an evolutionary adaptation enabling energy conservation [22,41]. Furthermore, the physical nature of some symptoms may influence behaviour. For example, lameness is associated with a decrease in activity, with animals spending more time lying and grazing for shorter periods [42], mainly due to an attempt to alleviate the pain that is experienced when putting weight on a diseased hoof. However, for mastitis in dairy cows, the opposite has been observed, with cows suffering from mastitis tending to spend more time standing up, moving and eating [43,44]. These findings suggest that pain may also stimulate discomfort and restlessness, which could explain our findings regarding ewes with increased FECs tending to be more restless.

Despite the demonstrated potential for using PLF technology to monitor sheep behaviours to guide anthelmintic treatment decisions for individual sheep, questions remain regarding the practicalities of using sensors on individual small ruminants, largely due to their cost in relation to the potential economic benefit. At present, on-animal sensor technologies are predominantly used in the dairy sector, where the cost–benefit of their use is widely accepted to be positive, especially for enhancing reproductive performance [45]. PLF technology has potential benefits in the sheep industry, with recent research demonstrating that collar-attached accelerometers can be a practical and feasible method of monitoring commercial flock behaviour [46]. Currently, measures implemented for TST of sheep, such as BCS in ewes and growth rates in lambs, can be recorded cheaply, and any future PLF technology would need to outperform these methods to be a viable option. Considering that declining BCS and growth rates will only be detectable following the onset of GIN-induced production losses, there may be potential for PLF technologies to identify sheep in need of treatment before animal performance is negated, a trait which would be a major benefit for sensor-based PLF over production-based TST markers. Alternatively, flock-level sensors which monitor the behaviour of a proportion of a flock could be used as a proxy to evaluate whole flock health, although this would only be applicable for the targeted treatment (TT) of sheep rather than TST. According to Melville et al. [47], the growth rates of 20% of lambs in a flock can be an optimal measure to determine the whole flocks’ anthelmintic treatment requirements. If monitoring the behaviour of a proportion of a flock would be similarly sufficient to enable maximal benefits of sensor-based PLF technologies in sheep systems to identify disease early, their viability and cost-effectiveness would significantly increase.

## 5. Conclusions

This research found that FECs of housed periparturient ewes were associated with detectable variations in ewe behaviour. This indicates that PLF technologies have the potential to identify ewes requiring anthelmintic treatment against GIN and therefore could be the basis of future TST strategies. However, inconsistencies seen between this study and previous studies regarding associations between animal behaviour and GIN infection burdens indicate that numerous factors appear to influence animal behavioural response to GIN infection. This currently limits the applicability of behavioural data as a TST proxy for GIN in ruminants without further complex studies and dataset modelling. In addition, technical challenges to overcome include improving the practicality of first-generation on-animal sensor technologies in the sheep sector, in order to increase their current cost benefits.

## Figures and Tables

**Table 1:** Means and standard deviations of parasitological, behavioural and general measurements recorded during study.

Descriptive Statistics		Mean	S.D
FEC (EPG)		241.85	283.8
Number of daily lying bouts (n/d)		27.93	6.71
Lying bout duration (min/bout)		29.75	7.31
Daily lying time (min/d)		792.79	112.76
BCS		2.85	0.51
Age		3.13	1.05
Lamb birth weight (kg)	Single (*n* = 22)	6.22	1.03
	Twins (*n* = 32)	4.92	0.53

**Table 2:** Linear mixed models of the association between log FEC and behavioural measurements (number of daily lying bouts, lying bout duration and daily lying time) of periparturient ewes.

Behavioural Measurement (Unit)	Estimate	S.E	Z	*p*
Mean lying bout duration (min/bout)	−3.745	1.451	−2.582	0.010
Mean number of daily lying bouts (n/d)	3.323	1.337	2.485	0.013
Mean daily lying time (min/d)	9.763	23.673	0.412	0.680

**Table 3:** Multivariate linear mixed models of factors associated with periparturient ewe behaviour.

Dependent Variable	Independent Variable	Estimate	S.E	Z	*p*
Mean lying bout duration (min/bout)	Intercept	37.927	9.563	3.966	<0.001
	Log FEC	−3.784	1.526	−2.479	0.013
	Litter size (Twin)	−0.585	2.492	−0.235	0.814
	Litter size (Single)	0	-	-	-
	BCS	−1.138	1.834	−0.621	0.535
	Age	1.122	0.907	1.237	0.216
	Mean lamb birth weight (kg)	−0.082	1.193	−0.069	0.945
Mean number of daily lying bouts (n/d)	Intercept	22.631	8.545	2.648	0.008
	Log FEC	3.606	1.364	2.644	0.008
	Litter size (Twin)	0.203	2.226	0.091	0.927
	Litter size (Single)	0	-	-	-
	BCS	2.066	1.638	1.261	0.207
	Age	−1.21	0.81	−1.494	0.135
	Mean lamb birth weight (kg)	−0.78	1.066	−0.731	0.465
Mean daily lying time (min/d)	Intercept	747.646	148.199	5.045	<0.001
	Log FEC	9.032	23.651	0.382	0.703
	Litter size (Twin)	39.287	38.613	1.017	0.309
	Litter size (Single)	0	-	-	-
	BCS	41.456	28.415	1.459	0.145
	Age	−24.04	14.052	−1.711	0.087
	Mean lamb birth weight (kg)	−7.285	18.49	−0.394	0.694

## Data Availability

The data presented in this study are available on request from the corresponding author.
